# Entropy Determination of Single-Phase High Entropy Alloys with Different Crystal Structures over a Wide Temperature Range

**DOI:** 10.3390/e20090654

**Published:** 2018-08-30

**Authors:** Sebastian Haas, Mike Mosbacher, Oleg N. Senkov, Michael Feuerbacher, Jens Freudenberger, Senol Gezgin, Rainer Völkl, Uwe Glatzel

**Affiliations:** 1 Metals and Alloys, University Bayreuth, 95447 Bayreuth, Germany; 2 UES, Inc., 4401 Dayton-Xenia Rd., Dayton, OH 45432, USA; 3 Institut für Mikrostrukturforschung, Forschungszentrum Jülich, 52425 Jülich, Germany; 4 Leibniz Institute for Solid State and Materials Research Dresden (IFW Dresden), 01069 Dresden, Germany; 5 TU Bergakademie Freiberg, Institut für Werkstoffwissenschaft, 09599 Freiberg, Germany; 6 NETZSCH Group, Analyzing & Testing, 95100 Selb, Germany

**Keywords:** HEA, entropy, multicomponent, differential scanning calorimetry (DSC), specific heat

## Abstract

We determined the entropy of high entropy alloys by investigating single-crystalline nickel and five high entropy alloys: two fcc-alloys, two bcc-alloys and one hcp-alloy. Since the configurational entropy of these single-phase alloys differs from alloys using a base element, it is important to quantify the entropy. Using differential scanning calorimetry, c_p_-measurements are carried out from −170 °C to the materials’ solidus temperatures T_S_. From these experiments, we determined the thermal entropy and compared it to the configurational entropy for each of the studied alloys. We applied the rule of mixture to predict molar heat capacities of the alloys at room temperature, which were in good agreement with the Dulong-Petit law. The molar heat capacity of the studied alloys was about three times the universal gas constant, hence the thermal entropy was the major contribution to total entropy. The configurational entropy, due to the chemical composition and number of components, contributes less on the absolute scale. Thermal entropy has approximately equal values for all alloys tested by DSC, while the crystal structure shows a small effect in their order. Finally, the contributions of entropy and enthalpy to the Gibbs free energy was calculated and examined and it was found that the stabilization of the solid solution phase in high entropy alloys was mostly caused by increased configurational entropy.

## 1. Introduction

The conventional strategy of alloy design is based on the selection of one base element for the primary properties of a material, e.g., iron in steels or nickel in nickel-based superalloys. This base element dominates the chemical composition, usually representing more than 80 at. %, and relatively small amounts of other elements are added to modify the alloys’ properties [1,2,3]. Thus the regions next to a single element of multicomponent phase diagrams have been well investigated in the past.

A novel way of alloy design concentrates on the unexplored centers of phase diagrams, where the alloys consist of elements in near equiatomic ratios. Such an alloy was pointed out by Cantor et al. [1] in 2004, when the equiatomic system of Cr, Mn, Fe, Co and Ni was found to result in a single-phase material. This simple face-centered cubic microstructure is free of any precipitates and stable over a wide temperature range [4]. Merely after long time annealing for 500 h at intermediate temperatures precipitations may segregate, e.g., a Cr-rich phase at 700 °C and three different phases (NiMn-rich, FeCo-rich, Cr-rich) at 500 °C [5]. However, a single-phase microstructure is the key factor for a high configurational entropy. In the same year Yeh et al. [3] pointed out the concept of high entropy alloys, independent of Cantor’s work. His definition of high entropy alloys is to consist of at least five or more elements with concentrations of each element between 5 and 35 at. % [6].

Many elements, and therefore a high value of configurational entropy, can lead to a more stable solid-solution phase with randomly distributed atoms [3]. A solid-solution phase with statistically distributed atoms in the crystal lattice is claimed to lead to interesting and outstanding properties, e.g., high hardness, wear resistance, high temperature strength and stability, sluggish diffusion, oxidation and corrosion resistance [6,7]. 

Yeh et al. [8] named four core effects, which are characteristic for microstructures and properties of high entropy alloys: the formation of one random solid-solution phase to reach a high entropy effect [8]; severe lattice distortion in the random solid-solution [9]; sluggish diffusion kinetics [6,10]; the so called “cocktail effect” [8]. 

Nevertheless the major part of investigated alloys with compositional requirements of high entropy alloys do not form single solid-solutions, but consist of several, mostly intermetallic phases, which can be brittle, difficult to process. This observation particularly disagrees with the crucial issue of a single-phase microstructure. 

Numerous examinations deal with the prediction of the conditions when a solid-solution phase is stable or additional intermetallic compounds are forming [7,10,11,12], but no reliable approaches have yet been proposed. Thus we follow the idea to calculate the total Gibbs free energy of an alloy system in single-phase state to compare it with formation enthalpies of several intermetallic compounds. Therefore the determination of thermal enthalpy and especially of entropy over a wide temperature range is necessary. A short insight in thermodynamics and in the way of calculating different parts of the entropy is given in the following part of the introduction:

The terms and definitions of the entropy theory, as well as the basic approach of determinations refer to Gaskell [13]. Changes in Gibbs free energy ∆G_total_ of a system at any state and temperature depends on the entropy S_total_ and on the enthalpy H_thermal_ (see Equation (1)). The consideration of both thermochemical parameters leads to a description of the equilibrium state of an alloy system by minimization of the Gibbs free energy at a fixed temperature:

(1) $$\Delta G_{\mathrm{total}}=\Delta H_{\mathrm{thermal}}-T\Delta S_{\mathrm{total}},$$

The entropy of an alloy consists of the configurational entropy S_conf_ [7] and the thermal entropy S_thermal_ [13]:

(2) $$S_{\mathrm{total}}=S_{\mathrm{conf}}+S_{\mathrm{thermal}},$$

(3) $$\Delta S_{\mathrm{conf}}=-R\cdot \sum_{i=1}^{n}x_{i}\cdot \ln x_{i},$$

The calculation of configurational entropy, also called mixing entropy, is given in Equation (3) with n as the number of elements, x_i_ the concentration of each element i and R as the universal gas constant. The equation is derived from the mixing of noble gases and is adopted to fully disordered solid solution, which are assumed in our work. If elements are distributed non-equally between possible sub-lattices, then a more general equation should be used [14]. In case of an equiatomic alloy, Equation (3) is reduced to $\Delta S_{\mathrm{conf}}=R\ln n$. Thus the configurational entropy of 5-component equiatomic alloy is ~1.6·R. At 0 K the resulting total entropy may not be zero. This does not violate the third law of thermodynamics (${\frac{\mathrm{dS}}{\mathrm{dT}}|}_{T=0}=0$) which is often misinterpreted as S_(T = 0)_ = 0. Crystalline solids may exhibit a non-zero entropy at the absolute zero point due to a randomly crystallographic orientation. A change of entropy in this point is not possible, because there is no ability of motion or diffusion.

The thermal entropy S_thermal_ can be directly determined by measuring the temperature dependent heat capacity at constant pressure c_p_(T) by differential scanning calorimetry (DSC):

(4) $$\Delta S_{\mathrm{thermal}}\left(T\right)=\int_{0}^{T}c_{p}\left(T\right)\frac{1}{T}\mathrm{dT},$$

Next to the entropy, there is an enthalpy-contribution to the Gibbs free energy, mentioned in Equation (1). This thermal enthalpy can be calculated using Equation (5):

(5) $$\Delta H_{\mathrm{thermal}}\left(T\right)=\int_{0}^{T}c_{p}\left(T\right)\mathrm{dT},$$

Therefore, the total change in Gibbs free energy at a certain temperature is presented in the following Equation (6). Setting the value of configurational entropy to zero, just the thermal entropy is considered and the result is the change in thermal Gibbs free energy:

(6) $$\Delta G_{\mathrm{total}}=\Delta H_{\mathrm{thermal}}-T\Delta S_{\mathrm{total}}=\int_{0}^{T}c_{p}\left(T\right)\mathrm{dT}-T(\Delta S_{\mathrm{conf}}+\int_{0}^{T}c_{p}\left(T\right)\frac{1}{T}\mathrm{dT}),$$

**Figure 1:**
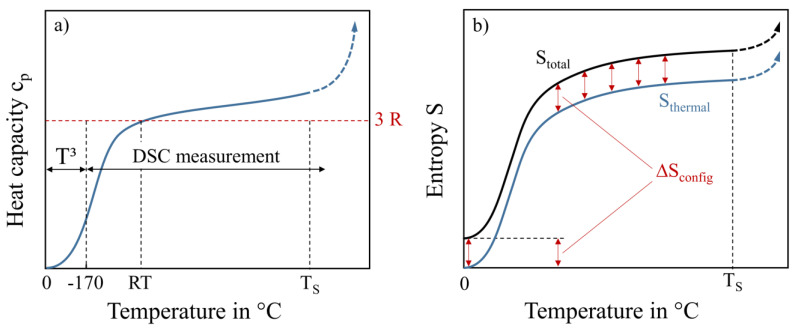
Schematic drawing (**a**) of the heat capacity of an ideal metal from 0 Kelvin to the liquid state with no change in magnetic behavior and no phase transition over the whole temperature range and (**b**) of the calculated thermal entropy and total entropy from 0 K to the liquid state.

A schematic drawing of c_p_ from 0 K to temperatures in the range of incipient melting after reaching the solidus temperature T_S_ is given in Figure 1a. For lower temperatures c_p_(T) can be extrapolated down to 0 K following the Debye T^3^-law [13]. For temperatures close to room temperature (RT) the heat capacity is close to 3R if there is no change in magnetic behavior and no phase transformation. This is known as the Dulong-Petit law, which states that every solid that consists of N atoms has 3N modes of vibration (corresponding to the freedom of motion in three dimensions). Energetic considerations using the equipartition theorem lead to c_p_ = 3·R for sufficiently high temperatures (RT). DSC measurements are carried out until the solidus temperature is reached and the c_p_-value rises dramatically. In Figure 1b, the calculated thermal entropy and total entropy are schematically illustrated. The thermal entropy is shifted vertically by the configurational entropy over the whole temperature range, in case there are no contributions by phase changes. This is due to the low influence of various alloys on the values of thermal entropy, while the configurational entropy has a high impact. The vertical offset between thermal and total entropy, caused by the alloys’ configuration, is still holding on after melting in the liquid state.

## 2. Materials and Methods

The temperature dependent molar heat capacity c_p_(T) of the specimen was experimentally determined in alumina crucibles using differential scanning calorimetry (DSC 204, Netzsch, Selb, Germany) in the temperature range from −170 °C to 600 °C under a flushing flow of nitrogen. In this temperature range oxidation of the samples is not critical. For T = 20 °C − T_S_ (if T_S_ < 1600 °C) samples were measured using a Netzsch DSC 404 F1 Pegasus under an Ar 5.0 gas flush with a rate of 70 ml/min in crucibles composed of 80% Pt and 20% Rh. The crucibles are lined with ceramic inlays to prevent interaction of the metallic specimens with the Pt-Rh crucible. The calibration of temperature and enthalpy of the two Netzsch devices was performed using the calibration set 6.239.2–91.3 under the conditions of 10 K/min and a nitrogen flow of 40 mL/min. The two measurements were evaluated for each material and the curves were connected in the common temperature range from room temperature to 600 °C.

Single-crystalline (SX) nickel and SX-Cantor alloy (favored equiatomic composition of Cr, Mn, Fe, Co, Ni) were cast using a proprietary Bridgman investment casting furnace. Cylindrical specimens (Ø 5 mm, height 1 mm) were then cut out of the rods by electrical discharge machining (EDM). All other alloys were in poly-crystalline state. The bcc-alloys were provided by Senkov et al. [15], the noble metal alloy by Freudenberger et al. [16] and the hcp-alloy by Feuerbacher et al. [17]. The recast layer of the EDM samples was removed by etching and the base of all samples was finely ground with SiC paper up to 2000 grit to ensure good thermal contact to the DSC-sensor. All materials tested several times showed good reproducibility in their DSC-signal, even at different heating rates of 10 K/min and 20 K/min respectively.

Except for pure nickel all materials belong to the high entropy alloys group. They form a single-phase microstructure and contain at least four elements in desired, near equiatomic composition. The single-phase solid-solution has been confirmed by the authors using X-ray diffraction experiments. Homogenization of the samples was reached by very slow cooling rates after melting, except of fcc-noble metal that was annealed for 24 h at 1000 °C and bcc 5-component was annealed for 24 h at 1200 °C.

Although the ROM may only represent a very rough estimate of the melting temperature of the alloys, it has been applied for the bcc-5 component and hcp-alloys. The reasons are twofold: (i) the melting temperature of the bcc-5 component alloy cannot be experimentally determined with our set-up and (ii) we are facing significant reactions of the metals with the crucible during investigation and, therefore, the determined values would not reflect the samples under investigation.

The Mn content of 11 at. % in the Cantor alloy is due to the single-crystal investment casting process. Mn evaporates from the melt in the vacuum of 10^−2^ Pa of the Bridgman furnace during the slow withdrawal of the single-crystal.

## 3. Results and Discussion

**Figure 2:**
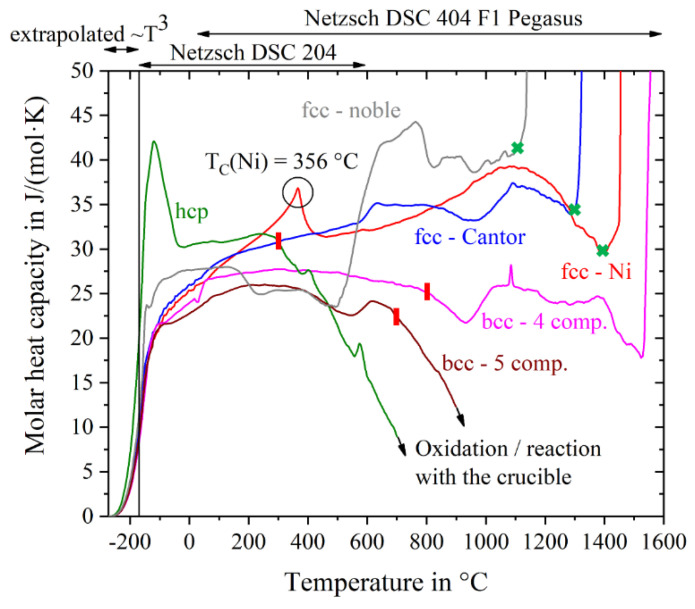
Molar heat capacities of pure nickel and five high entropy alloys. Green crosses indicate T_S_, red lines show the beginning of oxidation or reactions with the crucible that make the data not evaluable.

The specific heat capacity in units of J/(g∙K) has been converted into the molar heat capacity in units of J/(mol∙K) by multiplication with the molar mass of the alloys corresponding to their chemical composition. Figure 2 shows the molar heat capacity of all materials as a function of temperature. For temperatures below −170 °C, the curves were extrapolated using the fit-function a∙T^3^ (parameter a is listed in Table 1) and c_p_(0 K) = 0.

The two single-crystal fcc-materials, Nickel and Cantor alloy, show a steady increase in c_p_ until shortly before their melting points. Both curves are very close to each other with the exception of the Curie-peak in Ni at around 356 °C, which lies in excellent agreement with literature data (e.g., 354 °C [20]). The Cantor alloy shows a broad plateau-like peak between 600 °C and 800 °C. This peak is most likely caused by a change in magnetic behavior. Jin et al. [21] have shown that Co and Fe shift the Curie temperature of Ni to higher temperatures and their investigations on Cantor alloy show the exact same plateau-like peak starting at around 600 °C. Incipient melting at the solidus temperatures is indicated by excessive jumps in the curves. The solidus temperatures of fcc-nickel, fcc-Cantor and fcc-noble do not correspond to the values listed in Table 2, but differ by about 30 K in case of fcc-Cantor and fcc-nickel and by about 70 K in case of fcc-noble. The other three alloys show decreasing specific heat capacities at elevated temperatures, which indicates reactions with the atmosphere and/or the crucible. High chemical interaction and oxidation with the ceramic liners of the crucibles occur with the alloys Hf-Mo-Nb-Ti-Zr and Ho-Dy-Y-Gd-Tb, so their curves are finally cut-off at 700 °C and 900 °C respectively. Melting intervals, starting from the solidus temperatures, are not evaluable for the bcc-5 component and hcp alloy. In case of the bcc-4 component alloy the curve mistakenly suggests the solidus temperature to be at about 1520 °C. However, Senkov et al. [15] expect a melting temperature of about 2904 °C, using the rule of mixture. This issue is likely to be due to chemical reactions with the Pt-Rh crucible and therefore we have to regard the values of the heat capacity of bcc-4 component for higher temperatures with caution. For further calculations concerning entropy, enthalpy and Gibbs free energy, the data of bcc- and hcp-alloys respectively are just used in the temperature range until the red lines in Figure 2.

Table 2 gives an overview of all tested samples, their crystal structures, chemical compositions, configurational entropies and solidus temperatures. Note that in case of bcc-5 component and the hcp alloy, there was no literature data concerning solidus temperature available. Therefore, the possible solidus temperatures were calculated using the rule of mixture (ROM).

Obvious shifts in the trend of c_p_ indicate phase transitions or changes in the magnetic behavior, e.g., the Curie point of nickel at 356 °C as mentioned above. The hcp-alloy Ho-Dy-Y-Gd-Tb shows a very pronounced peak at −120 °C, probably due to a not investigated magnetic phase transformation, because oxidation seems to be very unlikely in such a low temperature range.

**Figure 3:**
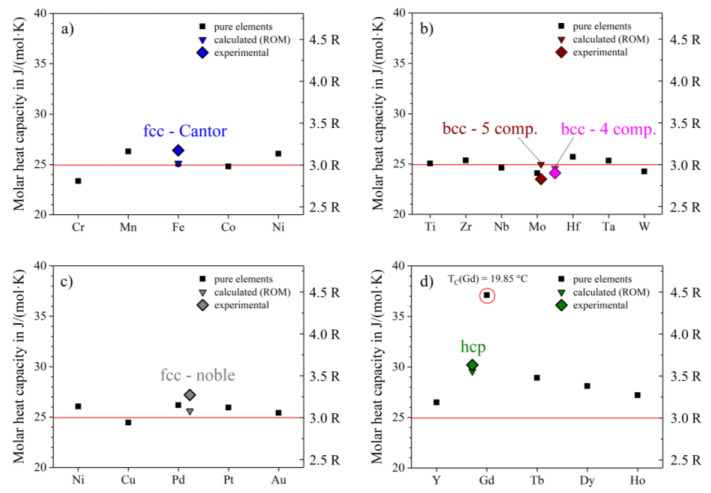
Molar heat capacities at room temperature of the investigated alloys, both experimental and rule of mixture (ROM) data for (**a**) fcc-Cantor, (**b**) both bcc-alloys, (**c**) fcc-noble and (**d**) the hcp-alloy. Values for pure elements were taken from [20]. The red line indicates the Dulong-Petit law with c_p_(RT) ≈ 3·R.

Of particular interest are the heat capacities at room temperature, where all curves seem to approach a c_p_ value close to 25 J/(mol∙K) = 3R (see Figure 3a–d), according to the Dulong-Petit-law [13]. The bcc-5 component equiatomic alloy, Hf-Mo-Nb-Ti-Zr, shows the lowest molar heat capacity at room temperature with about 23.5 J/(mol∙K) (Figure 3b), the hcp-alloy Ho-Dy-Y-Gd-Tb shows the highest value at about 30.6 J/(mol∙K) (Figure 3d). The high value of the molar heat capacity of the hcp-alloy might originate from magnetic ordering of the 4f electrons. This is likely the case as pure Gd shows ferromagnetic ordering below 19.9 °C. However, this suggestion needs to be verified in future studies. The other four alloys show RT c_p_-values pretty close to 25 J/(mol∙K). We applied the rule of mixture (ROM) to predict molar heat capacities of the alloys and their average molar mass (see Equation (7)). The calculated data is in very good agreement with the experimental results and shows a maximum deviation of 6%:

(7) $$c_{p,\mathrm{alloy}}=\frac{1}{n}\cdot \sum_{i=1}^{n}c_{p}{}_{i},\;\;u_{\mathrm{alloy}}=\frac{1}{n}\cdot \sum_{i=1}^{n}u_{i},$$

**Figure 4:**
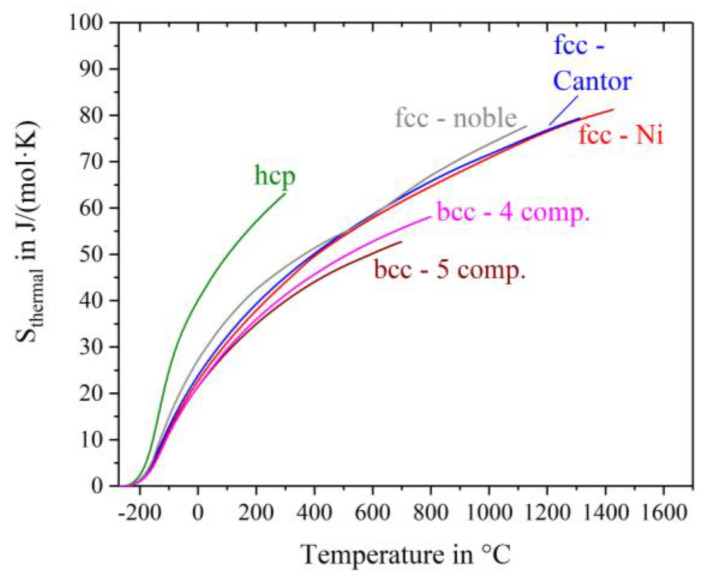
Thermal entropy S_thermal_ of nickel and the five high entropy alloys.

Figure 4 shows the thermal entropy S_thermal_ determined with Equation (4). S_thermal_ is equial zero at −273 °C andincreases continuously with increasing temperature having different slopes for different alloys. The curves are drawn till shortly before the solidus temperature of the alloys, except for Ho-Dy-Y-Gd-Tb, Hf-Mo-Nb-Ti-Zr and Mo-Nb-Ta-W where chemical reactions with the crucible/environment make the high temperature regions inaccessible. The highest thermal entropy appears for the hcp-alloy, caused by the highest heat capacity at low temperatures and additionally the early peak in the c_p_-curve at about −120 °C. The fcc-nickel, fcc-Cantor alloy and fcc noble metal alloy have almost the same temperature dependence of S_thermal_, which is noticeably weaker than that of the hcp-alloy. These fcc materials have similar S_thermal_ values at any given temperature. The two bcc-alloys exhibit the lowest values of thermal entropy.

**Figure 5:**
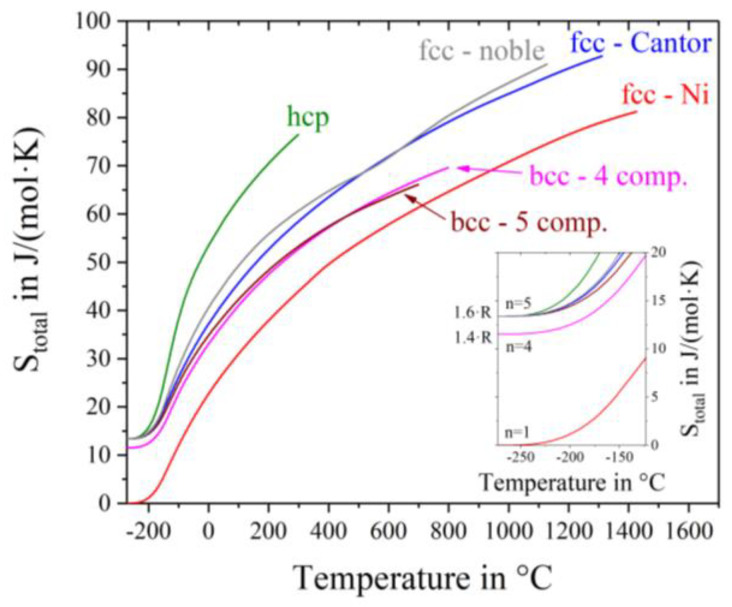
Total entropy as a sum of temperature dependent thermal entropy S_thermal_ and constant composition dependent configurational entropy S_conf_.

The total entropy S_total_ = S_conf_ + S_thermal_ over the whole temperature range starting from absolute zero is plotted in Figure 5. While the curve of nickel starts at 0 J/(mol∙K) at absolute zero, the bcc-4 component alloy Mo-Nb-Ta-W exhibits a configurational entropy of R ln(4) = 1.38·R = 11.5 J/(mol∙K), because of its four components. All other high entropy alloys, with five different elements show an offset of R ln(5) = 1.6·R = 13.4 J/(mol∙K). In theory the configurational entropy is equal for all alloys with the same number and concentrations of elements. No considerations are done with respect tosimilarities between participating atoms, like differences in the atomic radius or crystal structures of the pure metals. In our case, all elements in the hcp-alloy promote a hexagonal close packed crystal structure and exhibit quite equal atomic radii. The bcc-5 component alloy however consists of elements of different crystal structures with larger atomic size differences. Nevertheless, the same value of S_conf_ is assumed, as stated in the Gibbs paradox [22]. For dislocation movement, however, different atom sizes in solid-solution crystal structures indeed play an important role. Consequently, this mixing paradox will be investigated thoroughly in a future work. It can be seen from Figure 5 that at any given temperature S_total_ is the smallest for fcc-Ni, followed by the bcc alloys, then the fcc alloys and being the highest for the hcp alloy.

**Figure 6:**
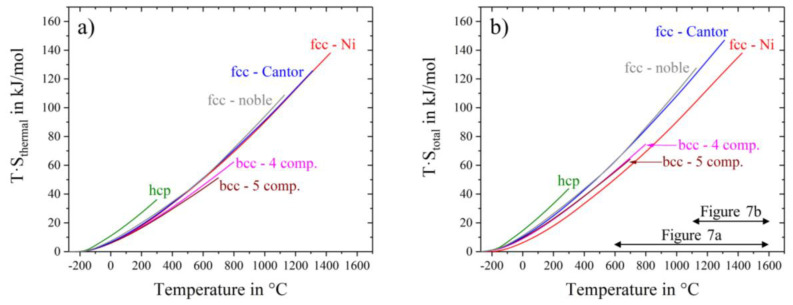
Contribution of thermal entropy (**a**) and total entropy (**b**) of nickel and all high entropy alloys to Gibbs free energy. The horizontal arrows refer to Figure 7 with magnified details of a special temperature range.

**Figure 7:**
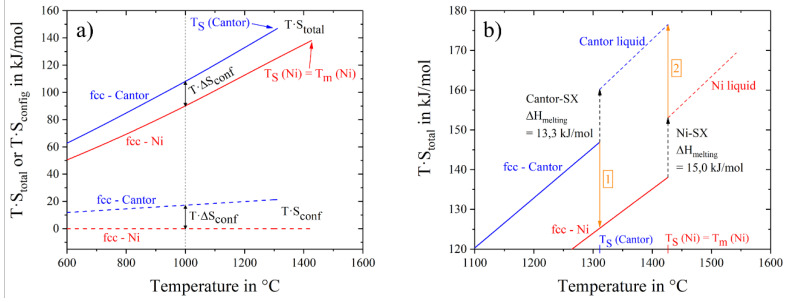
Contribution of total and configurational entropy of fcc-nickel and fcc-Cantor to Gibbs free energy in the range from (**a**) 600 °C to 1600 °C and from (**b**) 1100 °C to 1600 °C.

The contribution to the Gibbs free energy is the product of entropy and temperature. Using thermal and configurational entropy and their sum, we can examine their contributions to the Gibbs free energy separately. Figure 6a shows the energy contribution of thermal entropy. We can see a clear order with the alloy crystal structure: the highest contribution by thermal entropy is given by the hcp-alloy, mainly because of the high value of molar heat capacity at low temperatures (−120 °C) and also at room temperature. Pure nickel, noble and Cantor, all fcc-structures, overlie each other over a wide temperature range. The lowest heat capacities and therefore thermal entropy contributions are given by the bcc (4 and 5 component) alloys. Using the sum of thermal and configurational entropy, the total entropy times the temperature is shown in Figure 6b. Alloys with the same crystal structure and configurational entropy are close to each other. For example, the total entropy input to Gibbs free energy for fcc-Ni is smaller than for fcc Cantor and fcc nobel metal alloys. To explore the difference, two materials with fcc-structure, pure nickel and Cantor alloy, are drawn in Figure 7a in a temperature range from 600 °C to 1600 °C. Dashed lines show the configurational entropy contribution to Gibbs free energy of both materials, calculated by Equation (3). While the contribution of configurational entropy rises with higher temperatures for fcc-Cantor alloy, fcc-nickel does not exhibit any configurational entropy and, therefore, has no contribution to Gibbs free energy. Continuous lines show the product of the temperature and the total entropy. Thus the influence of configuration and thermal input can be quantified at certain temperatures. At 1000 °C the gap between continuous and dashed lines is almost similar, meaning that at this temperature there is a negligible difference in the influence of thermal entropy, but just the chemical composition of the solid-solutions affects the varying energy contributions. The energy level of total entropy contribution is about 90 kJ/mol for fcc-nickel and 108 kJ/mol for fcc-Cantor. The difference of 18.0 kJ/mol is very close to fcc-Cantor configurational entropy contribution of 1273 K∙R∙ln(5) = 17 kJ/mol (T∙S_conf_) at this energy level, resulting that thermal entropy has an equal impact for materials with the same crystal structures (in this case fcc) and thus cannot play role in stabilizing a solid solution phase in multicomponent alloys. Figure 7b shows a bigger section of the area near the melting interval of both materials. The two values of melting enthalpy 13.3 kJ/mol and 15.0 kJ/mol have been detected by DSC and it is obvious that differences in entropy contribution to Gibbs free energy still remains dependent from the configurational entropy until the end of the solid state. Investigations on the fcc-noble alloy instead of fcc-Cantor yield to similar results and are, therefore, not shown in Figure 7 in detail.

**Figure 8:**
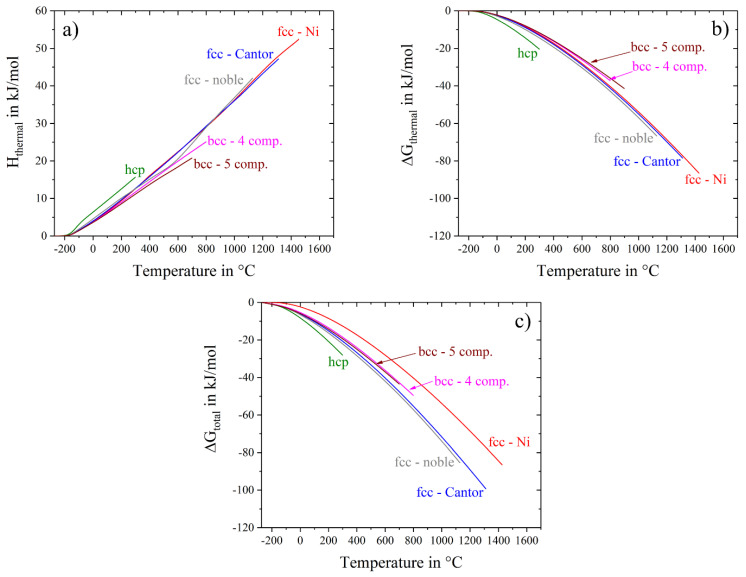
Temperature dependence of (**a**) thermal enthalpy, (**b**) thermal part of Gibbs free energy and (**c**) total Gibbs free energy for the studied materials.

Gibbs energy has another input besides entropy, namely thermal enthalpy, H_thermal_. The temperature dependence of the enthalpy, calculated with Equation (5), is displayed in Figure 8a, while Figure 8b,c show thermal Gibbs free energy and total Gibbs free energy, respectively. Similar to S_thermal_, H_thermal_ slightly depends on the type of the crystal structure having smallest values for the bcc alloys and highest values for the hcp alloy and shows almost no dependence on the number of components and alloy composition (Figure 8a). As a result of such behavior, the thermal part of the Gibbs free energy, ∆G_thermal_, also does not depend on the number and concentration of the alloying elements, but its temperature dependence is slightly stronger for the hcp alloy and weaker for the bcc alloys relative to the fcc materials (Figure 8b). On the other hand, the total Gibbs free energy, ∆G_total_, which additionally includes the configurational term, has a stronger temperature dependence and thus becomes noticeably smaller at higher temperatures for the alloys with larger configurational entropy (Figure 8c). These observations indicate that, although the contribution of S_thermal_ to the Gibbs free energy is much higher than that of than S_config_, at any given temperature ∆G_thermal_ is nearly the same for the simple and complex alloys of the same type of crystal structure, i.e., S_thermal_ does not play any role in stabilizing a solid solution phase in complex, multicomponent alloys: On the other hand, S_config_ increases with the number of constituents, which noticeably decreases ΔG_total_ of a multicomponent solid-solution relative to that of pure metals, especially at high temperatures. Moreover, in some specific cases ∆G_total_ of a multicomponent solid-solution with high S_config_ can become smaller than ∆G_total_ of competing intermetallic phases, resulting in a single-phase solid solution alloy. 

## 4. Conclusions

In this work the heat capacity, entropy, enthalpy and Gibbs free energy of six different single-phase solid solution alloys (three fcc-alloys, two bcc-alloys and one hcp-alloy) were experimentally determined and investigated over a wide temperature range.

- At room temperature (RT), molar heat capacities of the studied alloys are close to 3R, in accordance with the Dulong-Petit law.
- The measured RT heat capacities of the studied alloys are in good agreement with the heat capacities calculated using the rule of mixture of pure elements.
- Thermal entropy and thermal enthalpy increase while the thermal part of the Gibbs free energy decreases with an increase in temperature. The temperature dependence of these quantities is the strongest for the hcp alloy and weakest for the bcc alloys, with the fcc alloys showing intermediate behavior. For the alloys with the same crystal structure, the thermal contributions to the Gibbs free energy do not depend on the number and concentration of the alloying elements. Therefore, in spite the thermal entropy, S_thermal_, is much higher than the configurational entropy, S_conf_, at T > 20 °C, S_thermal_ does not increase thermal stability of a solid solution phase in complex, multicomponent alloys relative to simple alloys or pure metals.
- The compositional dependence of the Gibbs free energy of the solid solution alloys is totally due to the configurational entropy. Of the same crystal structure, solid solution alloys with higher S_conf_ have stronger temperature dependence of the Gibbs free energy and smaller ΔG_total_ values at a given temperature. Thus, although being smaller than S_thermal_, only S_conf_ contributes to the thermal stability of the complex, multicomponent solid solution alloys.

## Figures and Tables

**Table 1:** Values of the parameter a in J/mol∙K^4^ in the fit-function a∙T^3^.

hcp	bcc-4 Comp.	bcc-5 Comp.	fcc-Cantor	fcc-Nickel	fcc-Noble
1.8 × 10^−5^	7.6 × 10^−6^	8.2 × 10^−6^	9.8 × 10^−6^	9.0 × 10^−6^	1.0 × 10^−5^

**Table 2:** Sample labelling, crystal specification, chemical composition (measured by µ-XRF) and solidus temperature. In case of pure Ni, T_S_ is equal to the melting temperature T_m_. The rule of mixture (ROM) is used for calculation, if no literature data is available. All alloys are in a single-phase state over the entire temperature region.

Alloy Name Used in This Paper	Crystal Type and Structure	Chemical Composition (at. %)	S_conf_ in J/(mol∙K)	Solidus Temperature T_S_
fcc-Ni	SX-fcc	Pure Ni (>99.99%)	0	1455 °C [18]
fcc-Cantor	SX-fcc	Co_22_Cr_24_Fe_22_Mn_11_Ni_21_	1.58∙R	1280 °C [19]
fcc-noble metal	PX-fcc	Au_16_Cu_17_Ni_17_Pd_34_Pt_16_	1.56∙R	1196 °C
bcc-4 component	PX-bcc	Mo_27_Nb_27_Ta_23_W_23_	1.59∙R	2904 °C [15]
bcc-5 component	PX-bcc	Hf_21_Mo_20_Nb_21_Ti_17_Zr_21_	1.38∙R	2170 °C (ROM)
hcp	PX-hcp	Ho_18_Dy_16_Y_28_Gd_20_Tb_18_	1.61∙R	1416 °C (ROM)
